# Other Experiential Approaches Similar to Psychodrama

**DOI:** 10.1007/978-981-33-6342-7_15

**Published:** 2020-12-21

**Authors:** Scott Giacomucci

**Affiliations:** Phoenix Center for Experiential Trauma Therapy, Pennsylvania, PA USA

**Keywords:** Sociodrama, Sociatry, Drama therapy, Gestalt therapy, Experiential approaches, Teledrama

## Abstract

This chapter will briefly introduce further methods that Moreno created related to psychodrama, as well as other approaches similar to psychodrama but developed by others. Social microscopy and sociodrama will be outlined pertaining to Moreno’s additional dramatic approaches. Axiodrama, monodrama, autodrama, ethnodrama, bibliodrama, and teledrama each will be introduced as other dramatic approaches based on psychodrama. Drama therapy, gestalt therapy , Playback Theater , Theater of the Oppressed , Internal Family Systems Therapy, Pesso Boyden System Psychomotor Therapy, and family/systemic constellations will each be briefly presented with their similarities and differences to psychodrama.

Moreno is most recognized for his contributions to the field through his development of sociometry and psychodrama. Nevertheless, he also developed multiple other experiential approaches similar to psychodrama which are outlined below. Furthermore, other pioneers since Moreno have created experiential approaches have core elements in common with Moreno’s methods. Some of these other methods were directly or indirectly influenced by Moreno’s work while others emerged independently from his influence.

## Sociodrama

Sociodrama seems to be particularly complimentary to social work in its focus on social issues and versatility as an approach in clinical, educational, and community settings. Browne defines sociodrama as “a learning method that creates deep understanding of the social systems and social forces that shape us individually and collectively” (2011, p. 12). While a psychodrama enacts the topic or concerns of an individual group member, a sociodrama enacts a collective group concern (Giacomucci, 2017; Minkin, 2016). Moreno states that “the true subject of a sociodrama is the group” (1943, p. 437). In sociodrama, there is an element of aesthetic distance that helps maintain a sense of safety as the sociodrama is not an individual’s story; it is the story of the group. In a sociodrama, the group-as-a-whole is explicitly identified as the protagonist. The sociodramatic enactment “unlocks the common threads of human experience for everyone” (Sternberg & Garcia, 2000, p. xvii). Rather than sitting and talking about a social issue, group members take roles and enact scenes and themes related to the social issue. In doing so, group members are able to see the issue from the roles of others, clarify their values, and express their thoughts and feelings in a spontaneous manner. The goals of a sociodrama include catharsis and expression, insight and new perception, and role training or behavioral practice (Sternberg & Garcia, 2000).

A sociodrama session follows the same three phases of a psychodrama group—warm-up, enactment, and sharing. However, in a sociodrama, the enactment is not scripted in anyway and does not revolve around one person’s experience. Instead, the sociodrama scene is spontaneously put into action through co-creation between participants. Sociodramas tend to be more playful than psychodramas because of this and the aesthetic distance they provide. Sociodrama roles are focused on collective elements of the roles while psychodrama orients on the protagonist’s private elements of the role (Sternberg & Garcia, 2000). For example, in a group focused on parenting, a psychodrama would enact a protagonist’s parent–child relationship while a sociodrama would enact a hypothetical parent–child relationship. The role difference is—*your* child versus *a* child.

After a group warm-up identifying a central concern of dealing with a defiant child, the enactment phase of the sociodrama commences. The director invites two group members to take on the roles of a parent and a child while the audience is prompted to spontaneously define each of the roles in terms of age, gender, relationship, and other pertinent details. The group collectively defines the enactment which is put into action be the role-players. Once the originally identified concern of the group is embodied through the action, the facilitator pauses the action to ask for observations from audience members which allows them to reflect on the nature of the social interaction. Then the director invites doubling statements from participants for either the role of the parent or the child. Through doubling, the group begins to give voice to the internal experiences of both the parent and the child caught in this relational conflict characterized by the child’s defiance. New understandings emerge in this process that point to new ways of responding to a child’s defiance. The child’s defiance is re-enacted while participants take turns in the parent role offering new ways of responding. Once the director senses that the role training has been adequate, they move the scene to closure , de-roling, and finally sharing from the group.

The example above demonstrates a fundamental difference between psychodrama and sociodrama in that the scene is not scripted based on the protagonist’s experience—instead, it is spontaneously created by the group. In sociodrama, the themes, issues, roles, and scene can be defined by the group, the director, or a mix of the group and the director. In many cases, a sociodramatist is asked to facilitate a session with an already established contract or theme. A sociodrama can be person-centered or group-centered, but the roles remain collective instead of private roles (Garcia, 2011).

Because of its non-threatening but collective group focus, sociodrama is especially fit for education, professional training, community groups, conflict resolution, education, and social activism. Moreno described sociodrama as “a new approach to the problem of inter-cultural relations” in one of his earliest writings about it (1943, p. 434). He argues that social problems cannot be solved in the seclusion of individual therapy, but instead must be solved in a forum accessible to the entire community such as a sociodrama (1943). Sociodrama is frequently used in non-clinical settings and educational settings but can also be used in clinical groups (Giacomucci, 2017; Kellermann, 2007; Sternberg & Garcia, 2000). Sociodrama examples will be depicted in future chapters (especially Chap. 10.1007/978-981-33-6342-7_19) related to community work, social activism, and education.

## Social Microscopy and Sociatry

Moreno believed that “a truly therapeutic procedure must have no less objective than the whole of mankind” (1953, p. 1). All of his methods fall under the umbrella of his vision of *Sociatry*—healing for society (Moreno, 2006). He writes that “Psychiatry is the branch in medicine that relates to mental disease and its treatment; it treats the individual psyche and soma. Sociatry treats the diseases of inter-related individuals and of inter-related groups” (Moreno, 1947, p. 11). Sociatry, in practice, orients itself with Moreno’s mystical tradition and focuses on the larger societal picture and social justice (Giacomucci, 2018; Schreiber, 2018a).

The *social microscope* is a group, or societal, technique that Moreno developed to explore the invisible social forces that impact groups and society. Moreno comments that:

most sociodynamic phenomena disclosed by sociometry and sociatry “are” unconscious. But not unconscious in the sense of psychoanalysis, as repressed aggressive tendencies for instance, but unconscious almost in the sense in which the arrangements of the astronomic world were unconscious to man before he was able to study the stellar movements by means of scientific instruments. There are millions of atomic items buried in the group structures of human society which no human genius could divine and which no psychoanalysis of an individual mind lasting a thousand years could disclose. (1947, p. 22)

He wrote that these unseen forces “operate first in groups on the micro-sociological level then spread into the macro-sociological, leading to ever-larger ones” (Moreno, 2006, p. 514). Moreno created the social microscope in 1935 to explore how smaller groups are impacted by various psychosocial dynamics, believing that it could provide us with insight into how the larger society is impacted by the same dynamics. The social microscope makes visible the parallel process between group and society (Giacomucci, 2019). As Edward Schreiber says, “the group becomes a social microscope to the world sociometry” (2018a, p. 24). Moreno believed that we cannot prescribe a treatment for society, or for a group, if we do not understand the sociodynamics and organization of the group (Moreno, 1953). “Human society has an atomic structure which corresponds to the atomic structure of matter. Its existence can be brought to an empirical test by means of social microscopy” (1953, p. 697).

The social microscope is an experiential “instrument designed to illuminate sociatry” (Schreiber, 2018b, 18). This process uses a specific prescription of sociometric tests guided by Moreno’s developmental theory (Schreiber, 2017). Participants are warmed up to Moreno’s mysticism and engage in a hands-on-shoulder sociogram for each phase of the developmental theory—double, mirror, and role reversal—reflecting the phases of human development. With each new constellation of choices, the distribution of choices and connections is interpreted with emphasis on the sociocultural roles and identities of participants within the context of the current socio-political climate in society and the world. The following example comes from the ASGPP conference workshop.

After a series of warm-ups related to Moreno’s concept of the godhead, the encounter symbol, and the autonomous healing center within, the facilitator offers a prompt to participants, “put your hand on the shoulder of the person in the room who you experience as giving voice and expression to something that is already within you”. Participants move to make their choice creating a distribution of choices. The constellation results in the youngest group members chosen by the group as social stars and the older group members isolated with less choices. The facilitator describes this occurrence and suggests its connection to the importance of youth in society today giving voice to the injustices in society and how they need to be taken more seriously. At the same time he suggests that the elders being unchosen in the group may also reflect society’s lost sense of respect for elders. Then the facilitator moves to the next prompt, “put your hand on the shoulder of the person in the group who you experience as a mirror, reflecting back to you something important to know and grow into”. Participants make their choices, resulting in the majority of choices going to the women and transgender participants in the group with the men on the outsides of the constellations without many choices. The director acknowledges this to the group, commenting on the importance of reflecting on the significant role of women and trans folks in society today and going forward. That perhaps it is time for men to move to the periphery while dismantling patriarchal systems and create space for women and trans folks to be at the center of society and politics. The group becomes mystified by the truths emerging from the social microscope as the final criteria is given—“put your hand on the shoulder of the group member who, if you were to role reverse and experience the world through their role, your understanding and view of the world would expand”. Again, participants indicate their choices, this time resulting in the people of color being highly chosen while white group members were unchosen. The facilitator reflects to participants the distribution of choices as it relates to the continued racial inequalities and white supremacy in American culture. The need for role reversal, inclusion, and social justice is emphasized while relating back to Moreno’s concept of the organic unity of mankind. Group members reported feeling a sense of wonder and awe with a newfound understanding and motivation to work towards a socially just society. The director stresses the significance of the revelations from the social microscope as the emergence of the Godhead within the group.

The social microscope highlights both the social atoms and the cultural atoms impacting the group dynamics or its “the socio-atomic organization” within the sociometric matrix of group members (Moreno, 1953). It is designed to uncover both the sociodynamic effect and the organic unity of mankind (covered in detail in Chap. 10.1007/978-981-33-6342-7_5). The sociometric constellations of the process offer an opportunity for co-creation between the group and the Godhead—pointing to the organic unity of mankind and shattering the illusion of separateness between humans and groups. This instrument provides the group with insight into the sociocultural forces that threaten the unity of society while also helping the group to access its own autonomous healing center—the capacity to heal itself. “Sociatry’s task is to awaken us to the autonomous healing center in a group and organization, and to plant that awakening within the sociometric fabric of society” (Schreiber, 2018a, p. 24).

## Other Morenean or Psychodrama Approaches

In addition to sociometry, psychodrama, and group psychotherapy, Moreno also developed other action methods. His psychodrama textbooks introduce ethnodrama , axiodrama , monodrama , autodrama, and the use of psychodrama with dance, music, radio, and cinema (Moreno, 1946). A number of other action methods later developed drawing from or integrating psychodrama as a foundation, including bibliodrama , Souldrama , Therapeutic Spiral Model , Relational Trauma Repair Model, teledrama, and other culturally specific psychodrama approaches such as Sambadrama (Brazil) and Vedadrama (India). The etymology of terms becomes especially relevant here as Moreno created multiple new terms to label his ideas or methods.

*Psycho*—having to do with the mental, psychological, or soul.

*Socio*—related to society and the social.

*Axio*—meaning worth, truth, or value.

*Mono*—indicating a single aspect or the number one.

*Auto*—referring to self.

*Ethno*—related to ethnicity, race, or culture

*Biblio*—referring to books or the Bible.

*Tele*—meaning “at a distance” or “from afar,” communication over distances.

### Axiodrama

Moreno writes that axiodrama was the first of his dramatic methods to develop in 1918, later followed by sociodrama (1921), and psychodrama in the 1930 (Moreno, 2019a). He describes axiodrama by using spontaneous drama for dealing with issues related to values—cultural, religious, spiritual, and ethical. Axiodrama is most similar to sociodrama. Moreno’s personal life and attraction to existential and religious philosophies played a major role in his development of dramatic methods. Marineau (2013) writes that psychodrama, sociodrama, and axiodrama are intimately linked through their enactments of individual life (psychodrama), community life (sociodrama), and existential or religious life (axiodrama). Most psychodrama and sociodramatic enactments have roles or aspects that are axiodramatic in nature such as personal values, spiritual roles, social ideals, and death. “Ideally, Moreno’s followers should all be axiodramatists, psychodramatists and sociodramatists” (Marineau, 2013, p. 24). Some have suggested that axiodrama is a natural fit for religious communities (Blatner, 1996) and organizations (Souza & Drummond, 2017).

### Monodrama

In a monodrama, the protagonist plays all of the roles of the drama without any auxiliaries. There is only one role player. This approach is often used in individual therapy due to the absence of other participants (Blatner, 2000). Most often in a monodrama, other roles are indicated through the use of empty chairs. Monodrama is a core technique in gestalt therapy (Blatner, 1996). Monodrama is also referred to as *bipersonal psychodrama* or *psychodrama a deux*—meaning it involves two people, the director and the protagonist in one-on-one sessions (Cukier, 2008; Knittel, 2009).

### Autodrama

The terms monodrama and autodrama are often confused and used interchangeably though they have a subtle difference. A monodrama has only one role player, the protagonist—but it is facilitated by another person, the director. In autodrama, the protagonist *is* the director. Simply put, an autodrama is a self-directed enactment. When a drama is self-directed and involving only one person, then it is both an autodrama and a monodrama. Autodramas are often useful for experienced psychodrama participants or practitioners seeking to engage in their own personal work in an efficient manner.

### Ethnodrama

The integration of a psychodrama or sociodramatic process with the content of ethnic or racial conflicts is referred to as an ethnodrama (Malaquias, Nonoya, Cesarino, & Nery, 2016). Moreno initially describes sociodrama as an effective method for intercultural relations (1943), later using the term ethnodrama (1953). While Moreno is one of the earliest writers to use the term ethnodrama, he appears to have written very little about it. Ethnodrama seems to have become much more popular in theater, anthropology, drama therapy, and research (Mienczakowski, 2001; Saldaña, 2005). Snow and Herbison (2012) introduced *Ethnodramatherapy* as primarily an integration of ethnodrama with drama therapy, but also borrows techniques from sociometry, psychodrama, and Playback Theater. Snow and Herbison base their approach on Mienczakowski’s definition of ethnodrama:

ethnodrama is explicitly concerned with decoding and rendering accessible the culturally specific signs, symbols, aesthetics, behaviours, language and experience of health informants using accepted theatrical practices. It seeks to perform research findings in a language and code accessible to its wide audiences. (Mienczakowski, 2001, p. 468)

Ethnodramatherapy work has been used in psychotherapy contexts, diversity training, public performance, research, and educational groups (Snow et al, 2017).

### ***Bibliodrama***

While similar to axiodrama or sociodrama , bibliodrama is uniquely different in that it focuses on using role playing to bring to life stories and characters from religious texts (Pitzele, 1998). Bibliodrama is often used in religious communities or educational settings—it is also applicable as a process for exploring meaning of other mythical or archetypal stories and legends (Blatner, 2000).

A bibliodrama may be based around a protagonist or not—but follows the same group phases as a psychodrama or sociodrama and integrates the same interventions (Pitzele, 1998). While bibliodrama primarily enacts the written scripture, it is also used to explore the unspoken or unwritten parts of religious texts. It provides participants with an experiential understanding of the relational dynamics and existential dilemmas outlined in religious history. Bibliodrama provides an avenue for participants to deepen their connection to characters from scripture.

## Teledrama and Telemedicine

With the increased accessibility, reliance, and sophistication with technology, a new way of using Moreno’s methods has emerged (Fleury, 2020). Teletherapy is simply the provision of therapy services through the Internet or phone. Teledrama describes a method for using action methods for psychotherapy, training, and coaching in an online video format (Simmons, 2018). The term teledrama was coined by Daniela Simmons, albeit many other psychodramatists have been using action methods online for several years (Farnsworth, 2017; Hudgins, 2017; Pamplona da Costa, 2005). She writes on teledrama’s Web site that “teledrama is a very important part of the future of action methods and it is a bridge between countries and cultures”. Perhaps teledrama and telemedicine in general are the fulfillment of Moreno’s vision of using technology to create a therapeutic experience for larger groups within society (Moreno, 1946; Pamplona da Costa, 2005).

Two major recent events are likely to contribute to a significant increase in the use of Moreno’s methods online for teletherapy and distance learning. The first major change was a 2019 change in the American Board of Examiners in Sociometry, Psychodrama, and Group Psychotherapy regulations for distance learning which now permit a portion of training hours to be accrued through distance learning. The second major event was the 2020 CoVid-19 pandemic which proved to be a spontaneity test and catalyst for many in the international psychodrama community to begin offering online therapy and training events using Moreno’s methods (Giacomucci, 2020; Mindoljević & Radman, 2020; Vidal & Castro, 2020).

In the larger culture of the psychotherapy field, it seems that teletherapy is likely to become more utilized, in addition to online education, supervision, and training. Moreno (2019a) describes three revolutions in psychiatry: (1) Philippe Pinel’s humane treatment instead of punishment for the mentally ill in the eighteenth century; (2) Freud’s re-conceptualization of mental illness as psychological instead of neurological at the beginning of the twentieth century; (3) Moreno’s introduction of group psychotherapy in the 1930. Perhaps the movement to teletherapy and online professional development is the fourth major revolution in the field of psychiatry.

Nearly 100 years ago, Moreno had introduced ideas of using radio, film, and technology to create healing experiences for large groups of people—he used audio recordings in his 1930 Sing Prison work and video recordings from his work in Hudson New York at the girls training school. Around 80 years ago, Moreno founded a company called Therapeutic Motion Pictures with the hopes of providing healing and role training to larger audiences (Moreno, 2014 ). Jonathan Moreno, in his book recounting his father’s history, quotes from an unpublished Moreno paper in the 1940 which states:

The day will come when the engineer will provide us with a ‘two-way’ television system… every tele spectator will be able to televise himself back and so establish a communication between the therapist and himself multiplying the potentialities of a visual telephone by millions (Moreno, 2014, p. 257).

It seems that Moreno had envisioned the live video calls used for teletherapy today. I expect that he would have been excited at the evolution of teletherapy and online education as methods that meet people where they are and offer potentialities of large-scale therapeutic experiences. It is likely that Moreno would have also been pleased by the name “teletherapy” or “telemedicine” as it is related to his concept of “tele” (see Sect. 10.1007/978-981-33-6342-7_5). The psychodramatic concept of tele is defined as an accurate and reciprocated knowing or experiencing of between two people. Tele is seen as an interpersonal phenomenon necessary for the success of all relationships, including the working relationship between client and therapist (Z. T. Moreno, 2000). In a Morenean sense, one might argue that all therapy is “teletherapy.”

## Other Approaches Similar to Psychodrama

Beyond Moreno’s methods and modified psychodrama approaches are multiple other methods that developed with considerable overlap to psychodrama. Some explicitly trace their history back to Moreno while others use methods Moreno developed without much or any reference to him.

### Drama Therapy

While the term drama therapy is sometimes used interchangeably with psychodrama in the literature, it is its own unique field and approach separate from psychodrama with its own professional society, journal, degree programs, and credentialing board. Nevertheless, many drama therapists consider Jacob Moreno to be the first drama therapist (Bailey, 2006). There may be far more similarities than differences when it comes to psychodrama and drama therapy. Both are used as approaches in psychotherapy, education, and community work integrating role theory, drama, improv games, role playing, symbolism, spontaneity , creativity, and a biopsychosocial perspective.

In terms of differences, psychodrama focuses on enacting one individual’s story or topic while drama therapy enacts stories or topics related to the group-as-a-whole (similar to sociodrama). Psychodrama is more structured while drama therapy is more fluid. While psychodrama uses imagination and reality, drama therapy is more focused on symbolic or surplus reality. Psychodrama can be traced back *solely* to Moreno’s theoretical and philosophical foundation in the early 1900. On the other hand, drama therapy emerged with multiple different theoretical and practical approaches developed by different pioneers several decades after Moreno (Johnson & Emunah, 2009; Landy, 2017). Kadem-Tahar and Kellermann (1996) offer an eloquently stated differentiation below:

we have found that there is a fundamental difference between psychodrama and drama therapy. It seems that whereas in psychodrama the “soul” (psyche) is the aim and the “action” (drama) is the means, the opposite is true for drama therapy in which drama itself (as pure art) is the aim and the psyche is the means (of expression) (Kedem-Tahar & Kellermann, 1996, p. 29).

Drama therapy is more connected to theater and sometimes moves from a therapeutic process to a focus on creating a theater production (Landy, 2017). While some psychodramatists are also theater professionals, most are not. Interestingly, the field of drama therapy seems to have professionalized and integrated within academia, research, and higher education in the USA far more than psychodrama has. Multiple graduate degrees are offered in drama therapy in the USA while there is not a single graduate program in psychodrama.

Learning psychodrama is a required part of drama therapy education, as such, psychodrama interventions become a part of every drama therapist’s toolbox in an explicit way. Alternatively, psychodramatists do not all learn drama therapy interventions, though many psychodramatists are also drama therapists and some drama therapy techniques, especially warm-up games, have become integrated into the psychodrama culture.

### ***Playback Theater***

Playback Theater emerged in the early 1970, developed by Jonathan Fox who was experienced in psychodrama (Blatner, 2000; Fox, Fox, Salas, & Sparrow, 2000). Fox explicitly credits psychodrama for the foundation of Playback Theater and even notes how Playback Theater was largely developed on the original Moreno psychodrama stage in New Paltz, NY (2018). Playback Theater utilizes a small group of trained actors to spontaneously enact personal stories from the audience (Fox, 1994 ). Jonathan Fox writes that he sees Playback Theater as more connected to Moreno’s Theater of Spontaneity than it is connected to psychodrama (Fox, 2004). Playback Theater seems to be more closely related to sociodrama and drama therapy than psychodrama in that it uses metaphor and symbolism with a focus on the drama more so than the individual’s story. Playback Theater is more concerned with the process of putting stories into action than using drama or theater as a means to uncovering solutions for personal or collective problems (Fox, 2004). In Playback Theater, the storyteller remains an audience member while in psychodrama or drama therapy they become an active role player. It is important to note that Playback Theater is not a psychotherapy, though sometimes used within therapy and often providing a therapeutic experience for audiences.

Playback Theater is often integrated into psychodrama work and often is used in major events at the national psychodrama conferences. Playback Theater and Theater of the Oppressed are sometimes confused with each other though they also have fundamental differences. Playback emerged from Fox’s experience in theater and psychodrama while Theater of the Oppressed developed from Augusto Boal’s socio-political experience in Latin America.

### Theater of the Oppressed

While Playback Theater is focused on personal changes or revolution, Theater of the Oppressed uses the individual’s story as a catalyst for social revolution and collective change (Weinblatt, 2015). Theater of the Oppressed focuses on developing solutions to social issues while Playback Theater is less solution focused (Fox, 2004). Theater of the Oppressed and Playback Theater are similar in their use of an individual’s story as the script for the enactment, which differs from sociodrama’s spontaneously emerging storyline. Augusto Boal developed Theater of the Oppressed in the 1970 in South America which evolved to include multiple theater modalities including forum theater, image theater, invisible theater, and the rainbow of desire (Boal, 2000, 2013; Feldhendler, 1993; Oliveira & Araujo, 2012). Perhaps one of the simplest ways of differentiating Moreno and Boal is to remember the paradigms from which they developed their ideas. Moreno was an existential psychiatrist while Boal was a Marxist playwright (Oliveira & Araujo, 2012). Feldhendler (1993) notes that Boal’s Newspaper Theater and Moreno’s Living Newspaper are nearly identical. Boal participated psychodrama groups in the 1960 but denies that psychodrama or Moreno had influence on his developing of Theater of the Oppressed. Later in 1994, Boal dedicates his Rainbow of Desire book focused on using theater in therapy to Zerka Moreno (Boal, 2013). Similar to Moreno’s trajectory, Boal’s work was initially based on social justice issues in society but later he developed an approach for psychotherapy (Boal, 2013; Feldhendler, 1993).

In Theater of the Oppressed, a scene is played out for the storyteller until the point of the central conflict, at which point audience members are invited to step onto the stage and offer experiential demonstrations of solutions or responses to the situation. This method is quite similar to psychodramatic doubling. As the name suggests, Theater of the Oppressed concerns itself primarily with issues of social justice and oppression. Later, when living in France, Boal developed methods for working with European bourgeoisie by conceptualizing mild neurosis as internal forms of oppression (Blatner, 2000). The experience of a Theater of Oppressed session provides audience members with new ways of confronting moments of oppression and injustice through the co-created process. Theater of the Oppressed, Playback Theater , drama therapy , and Moreno’s methods have much in common and are often integrated together by psychodrama practitioners.

### ***Gestalt Therapy***

Gestalt therapy is an existential therapy focusing on the whole person while incorporating here-and-now awareness, relational emphasis, and experiential techniques (Perls, 1969a ). Fritz and Laura Perls created gestalt therapy in the 1940 and put it forth in their 1951 book *Gestalt Therapy* (Perls, Hefferline, & Goodman, 1951). Similarly, to psychodrama, gestalt emerged from the rejection of aspects of psychoanalysis, with emphasis on relationships, and use of the empty chair with role playing. Gestalt therapy seems to have outdone psychodrama in terms of popularity in the USA and offers a modality framed as both an individual and a group approach. Gestalt therapy’s focus on individual therapy, while psychodrama is much more group focused, may be one of the reasons it has achieved and maintained popularity. At the same time, some group workers critique gestalt therapists for simply doing individual therapy in a group setting and being unable to engage the group-as-a-whole. From a psychodrama perspective, gestalt therapy is considered a monodrama where the client works one-on-one with the therapist without auxiliary egos—even in group settings (Yablonsky, 1976). The audience in gestalt therapy has very little involvement compared to psychodrama. Psychodrama seems to be much more action-based using an open stage while gestalt puts the client into the *hot seat* and uses more introspection.

Who created the empty chair technique? Gestalt therapists often claim it for their founder, Fritz Perls while psychodramatists insist that Moreno created it. There is much misunderstanding about this, and it seems Perls is frequently credited with the development of the empty chair because he made it popular and brought it into mainstream culture with his public demonstrations at Esalen Institute. Nevertheless, historical analysis reveals that Perls was a frequent attendee of psychodrama sessions in New York and later writes in his memoir, *In and Out of the Garbage Pail*, that Moreno and psychodrama had considerable influence on him (Berne, 1970; Blatner, 1996, 2000; Moreno, 2019b; Perls, 1969b; Yablonsky, 1976). Walter Truett Anderson, journalist and encounter group leader, recalls an encounter between Moreno and Fritz Perls in 1969 at a psychology convention where they presented on the same panel—Moreno publicly confronts Perls, “I don’t mind you stealing my stuff, but you should have stolen all of it”. Perls responded, “Ah, Jacob, Jacob, when will you just accept your greatness?” (Moreno, 2014, p. 218).

### Internal Family Systems

Internal Family Systems Therapy (IFS) conceptualizes the psyche as having multiple parts with a centralized self—the core essence of an individual (Schwartz, 1994). This perspective is quite similar to Moreno’s role theory, with the self having parallels to the Morenean concept of the autonomous healing center within (Longer & Giacomucci, 2020). Like gestalt and psychodrama, IFS uses a non-pathologizing person-centered approach with a primary goal of promoting further integration within the self and in the external environment. In IFS, parts are categorized as either *exiled* parts or *protective* parts. Furthermore, there are two types of protective parts—proactive *managers* or reactive *firefighters*. IFS places considerable emphasis on defense mechanisms and asking permission from parts before engaging with interventions.

Internal Family Systems Therapy (IFS) was developed by Richard Schwartz in the 1980–1990 integrating a mix of family therapy, systems theory, and parts work. In the development of IFS, Schwartz was influenced by Fritz Perls empty chair work and by his work with Virginia Satir, who had integrated Moreno’s methods into the family therapy field (Schwartz & Sweezy, 2019). IFS seems to be utilized primarily in individual work, but also adapted to group work. IFS and gestalt are similar in this way, and in that they both emphasize parts of self while exploring inner parts with mindfulness and interoception. IFS group work is much more similar to psychodrama than IFS individual work as parts are *externalized* in the group with role players, but in individual therapy parts are interfaced *within* the individual. Very little has been published about the connection between IFS and psychodrama, albeit many practitioners and trainers integrate both into their work. Rachel Longer and I published a recent short article outlining their similarities and how both IFS practitioners and psychodramatists could benefit from adapting aspects of the other’s approaches (2020).

### ***Pesso BoydenSystem Psychomotor Therapy***

The Pesso Boyden System Psychomotor (PBSP) Therapy, sometimes called p*sychomotor therapy* for short, was created by dance teachers Albert Pesso and Diane Boyden-Pesso in the 1960 in New York. PBSP is focused on providing experiential corrective experiences with idealized roles to reverse the impact of unmet needs from childhood (Winnette & Baylin, 2017). A PBSP session or *structure* appears to be quite similar to a psychodrama enactment as they both use role playing within a group to recreate scenes from the past and wished for scenes (Blatner, 2000). Similar to the psychodrama process, the PBSP approach follows the lead of the protagonist, constructs a dramatic scene, facilitates de-roling of role players, and finishes by sharing from participants in the group. PBSP’s *witness* role has many similarities to the mirror position in psychodrama or the observing ego role in the Therapeutic Spiral Model.

Psychomotor therapy has a similar process as psychodrama but also has its own terminology, theory, and training process (Pesso, 1969; Pesso & Crandall, 1991). It also is more explicitly trauma-focused and body-oriented than classical psychodrama. A psychomotor therapist uses *microtracking* to follow the subtle non-verbal communication from the protagonist (van der Kolk, 2014). The facilitation of psychomotor structures is more contained, scripted, and intentional than a spontaneous psychodrama enactment. Although the Pesso Boyden therapeutic approach seems to have limited research and publications, it has received an increased spotlight due to Bessel van der Kolk’s 2014 chapter on it in his best-selling book *The Body Keeps the Score* and his newly offered *experiential psychodramatic workshops* based on his training with Albert Pesso.

### Family Constellations and Systemic Constellations

Family constellations and systemic constellations therapy were developed by Bert Hellinger in the 1970s with a focus on the family ancestral system or other system such as an organization (Hellinger, 2003). The constellations therapy process revolves around one client, usually in a group setting, who chooses group members to hold the places of different family members, ancestors, or members of the system (Carnabucci & Anderson, 2012). Instead of being referred to as roles or auxiliary egos like in psychodrama, they are referred to as *representatives*. The place where the experience emerges is called the *field*, akin to the psychodrama stage. The client physically places representatives in the field and returns to their seat to observe. A constellations session is much less action-based or dramatic as psychodrama and focuses on information that emerges for the client or representatives through intuition, thoughts, impulses, body sensations, or energy. Rather than acting, doubling, or role reversal, the representatives are instructed to attune themselves to the field. The facilitator checks in with the representatives asking them to report what they are experiencing in the field and encouraging brief movements or statements. The process involves very subtle movement, periods of silence, and significant time spent with each representative attuned to their inner experience. Once an issue is concretized in the field between members of the system, the facilitator will instruct them to make simple gestures or statements to each other in attempts to move toward resolution while the client observes (Carnabucci, 2018). Constellations work can get to systemic issues in a streamlined manner but misses nuances of experience due to minimal verbal involvement (von Ameln & Becker-Ebel, 2020).

Family constellation sessions are based on three *orders of love* outlined by Hellinger—(1) every member has the right to belong to the family, (2) wrongs in previous generations will be redressed in future generations, and (3) people have rank according to who entered the system first (Hellinger, Weber, & Beaumont, 1998). Carnabucci (2018) comments on Hellinger’s work as being related to Moreno’s concept of tele and making it the central mechanism of change in an explicit way. Carnabucci and Anderson (2012) write four major differences between the two approaches: (1) psychodrama focuses on conscious reality while constellations work focuses on the unconscious and ancestral; (2) psychodrama auxiliaries are role trained by information from the protagonist while constellation representatives learn information about their character from their inner experience; (3) in constellation work, *resonating statements* are used in a way similar to doubling statements in psychodrama but much less frequent and usually provided by the facilitator; (4) the psychodrama enactment places the protagonist within the drama which explores elements of their life while the family constellation session is takes places within the field of one’s ancestry. Psychodrama enactments generally have a clearly defined scene while constellation work takes places without concrete context (Carnabucci, 2018). While clear differences exist between the two approaches, they also have much in common, including being experiential group methods focused on the person within their social environment and larger systems. Both approaches are based in comprehensive existential philosophical systems with emphasis on spirituality that include applications in psychotherapy and beyond (Carnabucci & Anderson, 2012).

## Conclusion

While Moreno’s triadic system of sociometry, psychodrama, and group psychotherapy offers a comprehensive approach for work with individuals, groups, and communities, much value is also added to the social worker’s repertoire from Moreno’s other methods and approaches similar to psychodrama which developed later. A brief introduction to the aforementioned methods, most of which seem to be influenced by psychodrama, demonstrates the richness of methods involving role playing in the larger field while providing a greater appreciation for Moreno’s influence. The approaches above are outlined in a way differentiating each from the other, however in practice these approaches are often blended together by practitioners. A thorough investigation of each of these models is beyond the scope of this publication but has been presented elsewhere in the literature.
